# The continual innovation of commercial PET/CT solutions in nuclear cardiology: Siemens Healthineers

**DOI:** 10.1007/s12350-018-1262-3

**Published:** 2018-04-10

**Authors:** Bernard Bendriem, Jessie Reed, Kathryn McCullough, Mohammad Raza Khan, Anne M. Smith, Damita Thomas, Misty Long

**Affiliations:** Siemens Healthcare GmbH, MI, Knoxville, TN USA

**Keywords:** MPI, Hybrid imaging, PET, CAD

## Abstract

Cardiac PET/CT is an evolving, non-invasive imaging modality that impacts patient management in many clinical scenarios. Beyond offering the capability to assess myocardial perfusion, inflammatory cardiac pathologies, and myocardial viability, cardiac PET/CT also allows for the non-invasive quantitative assessment of myocardial blood flow (MBF) and myocardial flow reserve (MFR). Recognizing the need for an enhanced comprehension of coronary physiology, Siemens Healthineers implemented a sophisticated solution for the calculation of MBF and MFR in 2009. As a result, each aspect of their innovative scanner and image-processing technology seamlessly integrates into an efficient, easy-to-use workflow for everyday clinical use that maximizes the number of patients who potentially benefit from this imaging modality.

**Electronic supplementary material:**

The online version of this article (10.1007/s12350-018-1262-3) contains supplementary material, which is available to authorized users.

## Introduction

Non-invasive cardiac imaging plays a major role in the assessment of patients with known, or suspected, CAD. In addition to evaluating the existence, extent, and severity of CAD, cardiac imaging provides risk estimates for major adverse cardiac events and guides therapeutic decisions.^1^

Currently there are several imaging modalities in clinical use, including: Single photon emission tomography (SPECT), Magnetic resonance imaging (MRI), Computed tomography (CT), and Positron emission tomography (PET). The latter captures the in vivo distribution of intravenously injected positron-emitting radiopharmaceuticals that reflect biochemical pathways of the target organ(s). Dependent on the injected radiopharmaceutical and selected protocol, PET is able to assess myocardial viability, myocardial perfusion, and various inflammatory cardiac pathologies. Cardiac PET is also able to provide absolute quantification of MBF and MFR.^1^ The ability to derive these values from non-invasive imaging represents a major breakthrough in cardiac imaging and demonstrates the potential of these measures to have a strong prognostic value. Most PET scanners combine with a CT to form a PET/CT hybrid device. The co-registered CT images provide anatomical information that the system uses for the attenuation and scatter correction of the PET data. In addition, the latest CT features enable non-invasive assessment of the coronary arteries and further enhance the PET information.^1^,^2^

According to the American Society of Nuclear Cardiology (ASNC) and the Society of Nuclear Medicine and Molecular Imaging (SNMMI), the following qualities emphasize the clinical usefulness of PET/CT in the management of patients with CAD.

- MPI PET/CT demonstrates a high diagnostic accuracy and provides consistently high-quality images. Also, compared to most diagnostic cardiac imaging modalities that utilize radiation, cardiac PET/CT has a relatively low radiation exposure.
- MPI PET/CT is time-efficient and, dependent on the injected radiopharmaceutical, a complete cardiac rest/stress study can be acquired in less than 1 hour. Typical acquisition times are 5 minutes compared to 1- to 2-day protocols with 20- to 30-minute acquisition times for rest and stress SPECT MPI.^1^,^3^
- The combination of MPI PET/CT with MBF quantification can further improve risk and treatment stratification, disease detection, and the ability to monitor lifestyle modifications or therapeutic interventions.^1^,^3^,^4^
- The capability to routinely and non-invasively quantify MBF in mL/min/gram is unique to PET/CT.^4^

An announcement from the American Medical Association (AMA) recognizes the increasing clinical importance of functional imaging and MBF quantification. The announcement regards the Current Procedural Terminology (CPT®) Editorial Panel’s approval of a category III billing code for PET-based absolute MBF quantification that took effect on January 1st, 2018.^5^ Given this recent reimbursement support and the aforementioned strengths of PET/CT, the clinical availability of MBF quantification is now poised to further play an important role in the evaluation of vascular dysfunction.^4^ A positive impact on CAD patient management in the next few years will facilitate the continued clinical adoption and growth of cardiac PET/CT.

The availability and regulatory approval of suitable radiopharmaceuticals are requirements for the clinical adoption of any PET- or SPECT-based technology. While different regions of the world have their own regulations, the following three tracers are currently approved by the Food and Drug administration (FDA):

- Fluorine-18 fluorodeoxyglucose (FDG) for viability and inflammation imaging
- Rubidium-82 chloride and Nitrogen-13 ammonia for perfusion imaging and estimating MPI and MBF^2^,^6^

Rubidium-82 chloride (produced in a Strontium-82 generator) and Nitrogen-13 ammonia (produced in an onsite cyclotron) show promising clinical results as both allow the application of compartment model kinetics to produce more reliable MBF quantification. (Table 1)

**Table 1:** Properties of radiopharmaceuticals used for cardiac PET/CT imaging

	Rubidium-82 chloride (generator)	Nitrogen-13 ammonia (cyclotron)	Oxygen-15 water (cyclotron)	Fluorine-18 flurpiridaz (regional cyclotron)	Fluorine-18 fluorodeoxyglucose (regional cyclotron)
Physical half-life time (min)	1.27	10	2.0	110	110
Typical dose stress/rest 3D PET mCi, (MBq)	30/30 (1110/1110)	10/10 (370/370)	20/20 (740/740)	6/2 (222/74)	N/A
Effective dose stress/rest per scan in mSv	1.3/1.3	1.0/1.0	0.7/0.7	4.4/1.5	3.5–10.5 (1 FDG injection)
Peak stress/rest extraction (%)	35/70	95/100	100/100	95/100	N/A
Peak stress/rest retention (%)	25/70	50/90	0	55/90	N/A
FDA approval	Yes	Yes, ANDA for onsite production^a^	No	In Phase III trials	Yes
Protocol particularities	Rapid protocol	Exercise possible; delay between rest and stress^b^	Rapid protocol^c^	Exercise possible; different doses rest and stress^b^	Requires optimizing of myocardial glucose utilization^d^
Prompt gamma emission	776 keV gamma rays	No	No	No	No

Properties of radiopharmaceuticals^2^,^3^,^8^,^39^

^a^Abbreviated New Drug Application

^b^Only for perfusion imaging, challenging for MBF

^c^No perfusion imaging due to missing retention

^d^Specifics in^2^

Although currently not approved by the FDA, Oxygen-15 water is perfectly suited for MBF quantification due to its 100% extraction rate.^3^ Fluorine-18 flurpiridaz, also not currently FDA approved, has the potential to overcome some of cardiac imaging’s limitations. Fluorine-18 flurpiridaz’s MBF quantification is expected to be very similar to Nitrogen-13 ammonia, and also potentially close to Oxygen-15 water, due to its high extraction fraction. It is an attractive radiotracer because, like FDG, it does not require an onsite cyclotron or generator for production and it yields high count images. Table 1 summarizes the main characteristics of the radiopharmaceuticals used in cardiac PET/CT imaging.

Widespread clinical adoption of non-invasive cardiac PET/CT depends not only on the growing evidence of impact on patient management and the expansion of clinical expertise, but also on the availability of this state-of-the-art technology. Since cardiac PET/CT MPI is technically challenging, it is critical that the clinical workflow be efficient and provides accurate and reproducible results. As a trendsetter in cardiovascular imaging, Siemens Healthineers offers a variety of PET/CT platforms that provide a complete set of tools to support a comprehensive clinical workflow for the assessment of cardiac diseases. These functionalities are accessible across all of the available Siemens Healthineers PET/CT products, thereby addressing all market segments.

## The Scanner: Biograph mCT

### Detection System

Siemens Healthineers utilize LSO scintillators in all of their PET detectors. As shown in Table 2, LSO offers the best combination of properties of any PET scintillator known today. Present-day PET/CT scanners now operate in 3D mode (without septa) and can register coincidence events between all possible detector rings. The resulting increase in sensitivity comes at the expense of more scatter, randoms, and dead time. In cardiac PET/CT, scatter and randoms have a tendency to artificially increase counts in areas with low uptake that are surrounded by higher activity, thereby making the identification of subtle perfusion defects challenging.^2^ The high light output and fast decay time of LSO facilitate better rejection rates of scatter and random events due to optimized energy discrimination and a fast coincidence timing of 4.5 ns. Existing 3D-vs-2D comparative studies show that Lutetium-based crystals, combined with improvements in electronics and software, can overcome the challenges of 3D-scanner acquisitions.^2^,^7^ The inherent properties of LSO utilized in Siemens Healthineers’ 4 × 4 mm crystals, paired with time of flight (ToF), translates to high spatial sampling and resolution as well as an improvement in image quality.

**Table 2:** PET detector material properties

Property	Characteristic	Desired outcome	LSO	BGO	GSO	NaI
Density (g/cc)	Defines detection efficiency of detector	High	7.4	7.1	6.7	3.7
Effective atomic number	Scanner sensitivity	High	65	75	59	51
Decay time (ns)	Influences detector dead time and randoms rejection	Low	40	300	60	230
Relative light output (%)	Impacts spatial and energy resolution	High	75	15	35	100
Energy resolution	Influences scatter rejection	Low	10	10.1	9.5	7.8
Non-hygroscopic	Simplifies manufacturing, improves reliability and reduces service costs	Yes	Yes	Yes	Yes	No
Ruggedness		Yes	Yes	Yes	No	No

LSO’s fast scintillation decay time of 40 ns reduces the detector’s dead time and enables the use of ToF information: a crucial advantage for imaging Rubidium-82 chloride studies. LSO’s high density ensures optimal detection efficiency. High relative light output allows the use of small detector crystals (4 × 4 mm) that provide the base for outstanding isotropic spatial resolution.^19^

Due to the combination of 3D-acquisition mode and ToF, Biograph mCT requires less injected activity which translates to a reduction in the patient’s effective dose. For short-lived tracers, such as Rubidium-82 chloride, adequate activities are needed to ensure satisfactory myocardial perfusion image quality within an acceptable acquisition time.^8^,^9^ A PET/CT system with the ability to perform a single-injection protocol for MBF and MPI—without detector saturation—is ideal as detector saturation causes an incorrect elevation of MBF values.^8^,^9^ To reduce the possibility of detector saturation an adequate dynamic range of the detection system, combined with the ability to obtain high-quality images with lower doses of Rubidium-82 chloride, is essential. Among ten commercially available systems, Biograph mCT provides the highest-tolerated maximal activity of 0.39 mCi/kg (14.4 MBq/kg) and is also one among four PET/CT scanners that is considered suitable for the clinically efficient single-injection protocol using 0.3 mCi/kg (10 MBq/kg) of Rubidium-82 chloride.^9^

### Gantry Configuration and Ease of Use

The body habitus of patients with CAD varies significantly and the industry-standard bore size of 70 cm is often inadequate to accommodate some patients. In addition, some patients can cause a vertical deflection of the bed that results in a misalignment of the PET and CT data. This misalignment can cause potential image artifacts due to AC errors.

**Figure 1:**
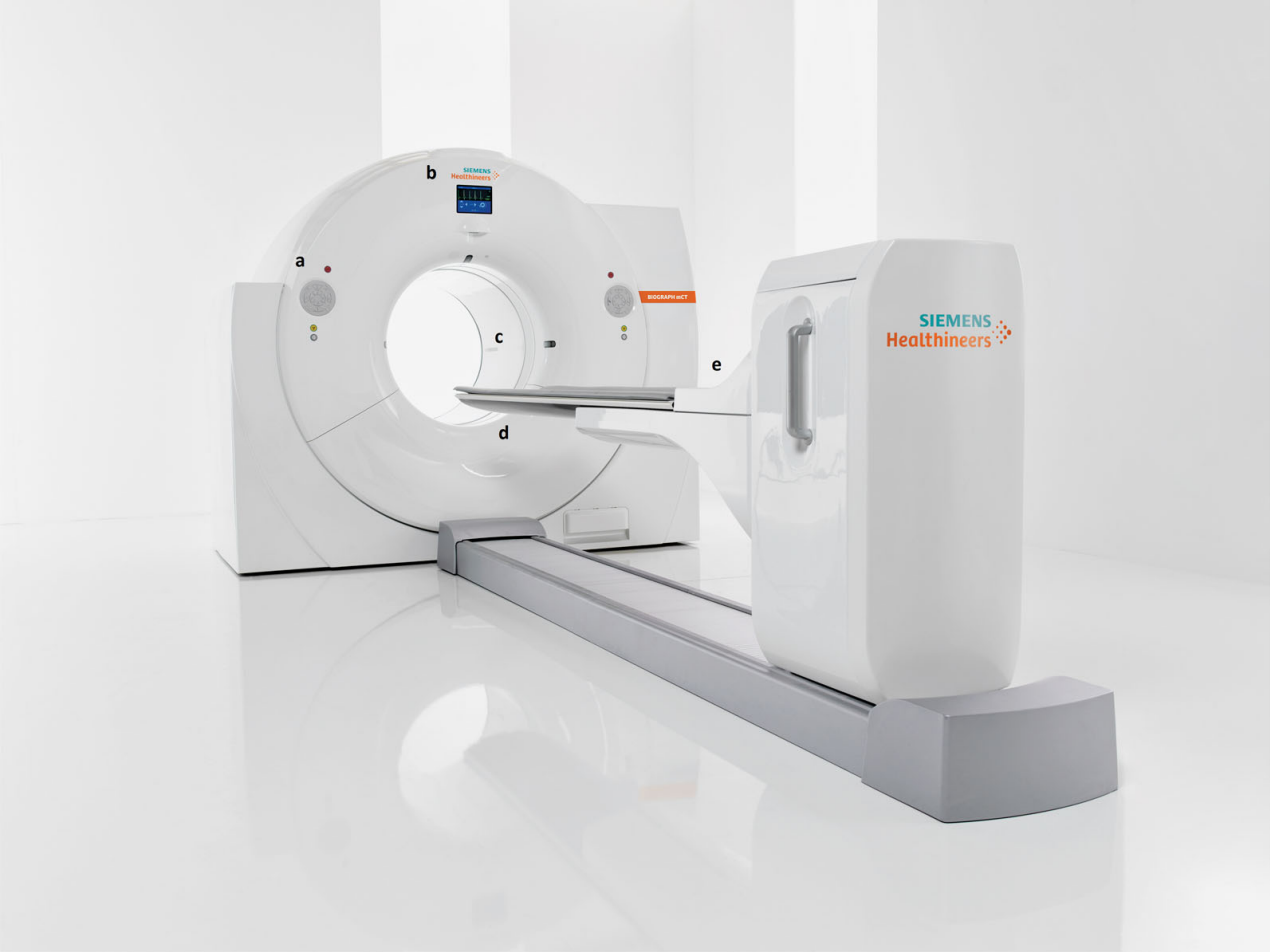
The scanner—Biograph mCT (a) convenient front-and-back access to the gantry control panels (b) a visual of the ECG signal on the gantry display panel (c) 78 cm bore width enhances patient comfort and supports the study of a broad patient population (d) state-of-the-art LSO scintillators ensure optimal image quality and signal processing (e) convenient ECG lead plug-in access within the PHS pedestal ensures secure equipment placement, patient safety, and patient comfort

For these patients, the 78 cm bore size of Biograph mCT is an advantage over the industry-standard 70 cm bore size. Combined with a short tunnel and a patient handling system (PHS) that supports up to 500 lb (227 kg), the wide bore enhances patient comfort and supports the study of a broad patient population. Due to the unique cantilever design of the PHS, the pedestal and the table move as one unit. Such movement results in zero differential deflection between the CT and the PET acquisition. This unique concept eliminates the risk of registration artifacts between the CT and PET due to table flexion (Figure 1).

For MBF studies, the PET acquisition must start before the injection bolus reaches the right ventricular cavity to ensure the system accurately captures the BIF.^10^ For most conventional PET/CT systems, this would require two technologists: one to start the acquisition and one to manage the radiopharmaceutical injection. To simplify the acquisition, Biograph mCT has control buttons on the front and the back of the gantry. This design enables one technologist to safely begin the acquisition and trigger the radiopharmaceutical injection simultaneously, thereby eliminating the need for additional resources and increasing optimal synchronization between injection and scan initiation.

### PET Image Formation

In a PET/CT scanner, each pair of parallel and opposite detectors produces a coincidence line, or line of response (LOR), that localizes a positron annihilation event along a specific line. A PET dataset is formed by a large number of LORs that reconstruct a cross-sectional image once they are fully processed.

**Figure 2:**
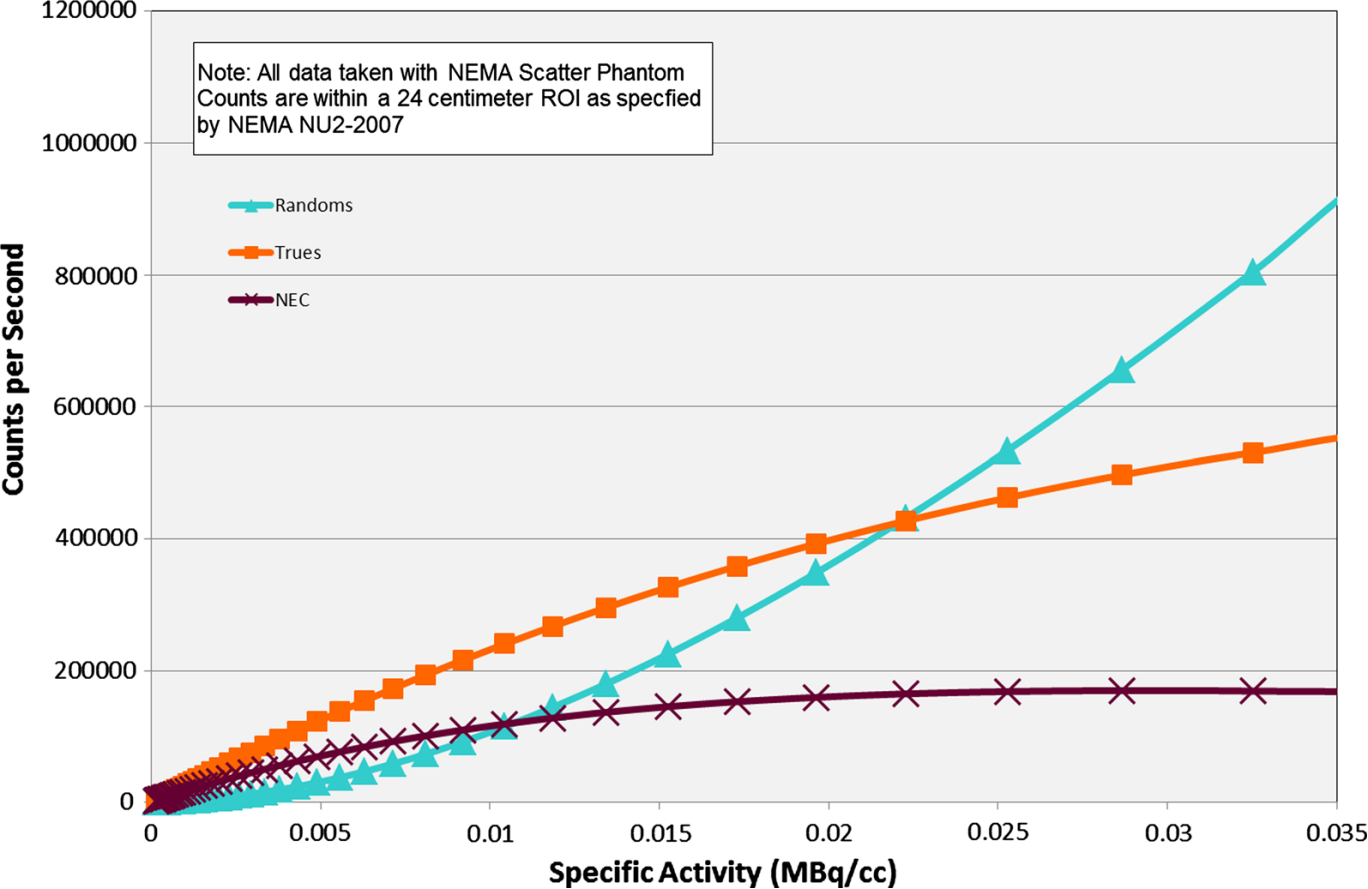
Count Rate Performance showing Biograph mCT TrueV reflecting the high count rate performance of the fourth ring. The increase in sensitivity may facilitate the use of lower doses of Rubidium-82 chloride in a combined MBF-MPI protocol

Biograph mCT’s optional fourth detector ring, TrueV, offers an extended field of view (FoV). With this extended FoV, Biograph mCT’s noise equivalent count rate (NECR) increases by 70% and delivers improvements in sensitivity and image quality (Figure 2).

Biograph mCT’s dedicated hardware and software tools, such as uniquely designed detector crystals, sophisticated coincidence electronics, and a diversity of signal-correction algorithms, help establish and reinforce the important role of cardiac PET/CT in clinical routine. Cardiac CT innovations complement Biograph mCT’s tools to offer optimal temporal and spatial resolution as well as flexible, yet straightforward, acquisition techniques.

### Data Processing

Rubidium-82 chloride is commonly used in clinical practice, yet the additional 776 keV prompt gamma ray emission produces significant background activity in the emission data. Without correction, the prompt gamma ray can result in loss of image contrast, increased noise, and quantitative errors.^11^ Biograph mCT’s PGC algorithm automatically addresses the impact of this non-pure isotope positron emission within integrated scatter correction.^12^,^13^ Such an improvement in image quality and quantification specifically supports prompt gamma isotope studies such as MPI and MBF examinations with Rubidium-82 chloride.

### CT Features

In addition to providing attenuation correction for cardiac PET imaging, the CT of Biograph mCT functions as a high-end, stand-alone CT scanner to non-invasively assess coronary artery morphology. High-quality cardiac CT requires optimal temporal and spatial resolution as well as flexible acquisition techniques. The CT has a gantry rotation time of 0.33 seconds which allows for virtually motion-free imaging of the heart. The Combined Applications to Reduce Exposure (CARE) feature includes CARE Dose and CARE KV which automatically adjust parameters to optimize image quality and reduce dose. CT bolus tracking and CARE contrast optimize the CT scan and contrast media injection, which increases process efficiency and standardization of care.

### SMART Auto Cardiac Registration

**Figure 3:**
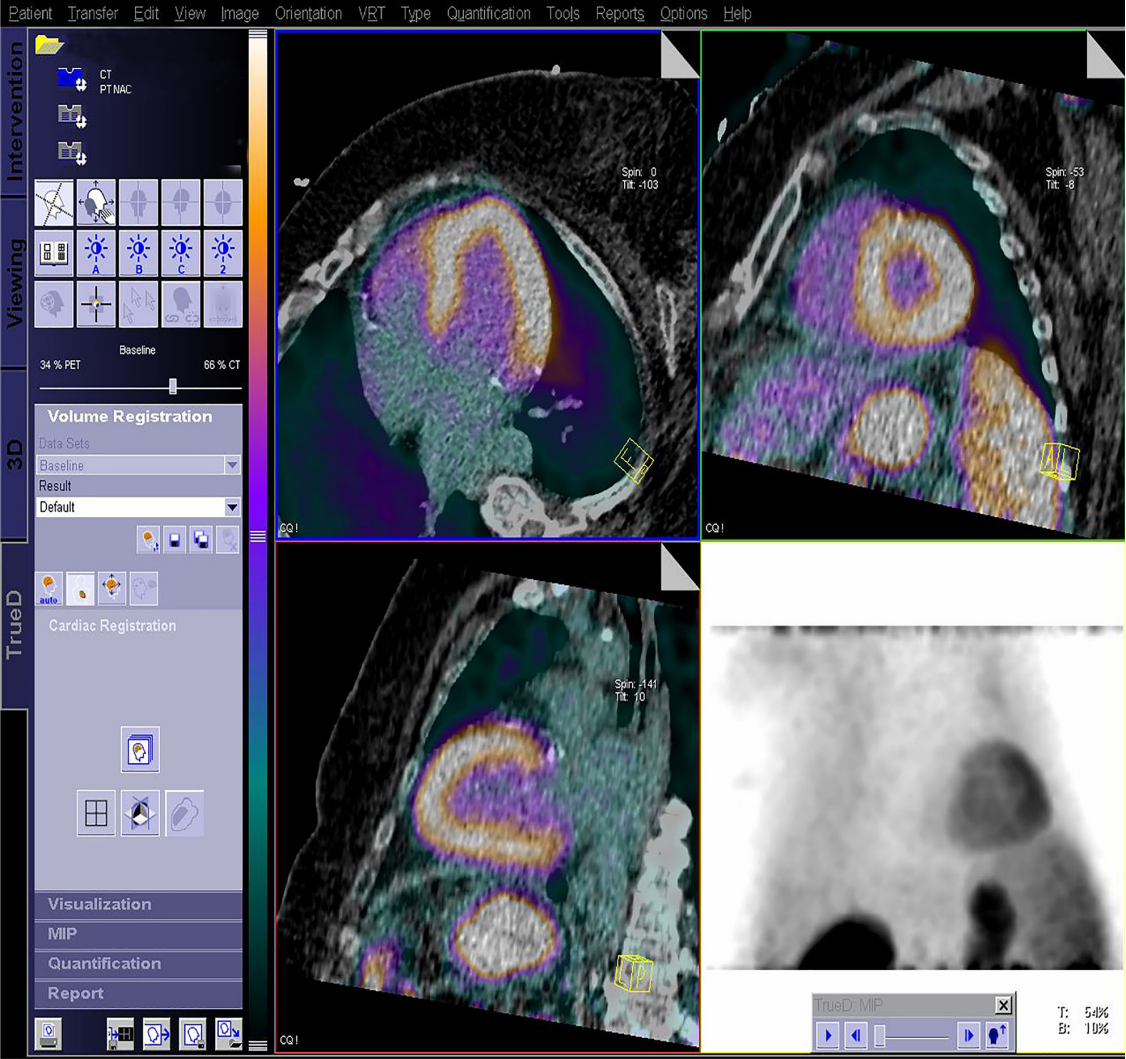
SMART Auto Cardiac Registration automatically aligns the non-attenuation-corrected (NAC) PET with the CT data in order to ensure optimal AC. The fused data is presented in transverse, coronal, and sagittal views. Data courtesy of University of Michigan, Ann Arbor, USA

Attenuation correction helps compensate for the attenuation of photons emitted by the human body, but requires an optimal alignment between the emission (PET) and the transmission (CT) scans. Due to the sequential nature of the acquisition protocol, there is a risk of misalignment between the functional PET and anatomical CT images of the heart. The resulting mismatch can ultimately affect the attenuation-corrected data: for example, by suggesting a false-positive perfusion defect.^14^ Conventional correction methods require the user to perform a manual alignment of the data, which is time consuming and often causes inter- and intra-user variability.^14^–^16^ In response, Siemens Healthineers developed an automatic registration algorithm: SMART Auto Cardiac Registration. SMART Auto Cardiac Registration integrates into the cardiac acquisition workflow and automatically registers cardiac PET and CT data for optimal attenuation correction. The algorithm focuses solely on the optimal alignment of the heart in both datasets (Figure 3).

SMART Auto Cardiac Registration shows optimal results using a translation-only registration algorithm.^17^ The algorithm was evaluated on 413 PET/CT image pairs and the results confirmed the robustness, consistency, and accuracy of the algorithm. The findings also underlined the algorithm’s potential to reduce the processing time and remove inter-operator variability.^14^ In a more recent review,^18^ the authors concluded that Siemens Healthineers’ registration solution significantly improves PET/CT AC alignment and the diagnostic accuracy of Rubidium-82 chloride PET/CT perfusion imaging when compared to data with no CT–AC alignment or with manual alignment.

### Optimized PET Image Reconstruction

**Figure 4:**
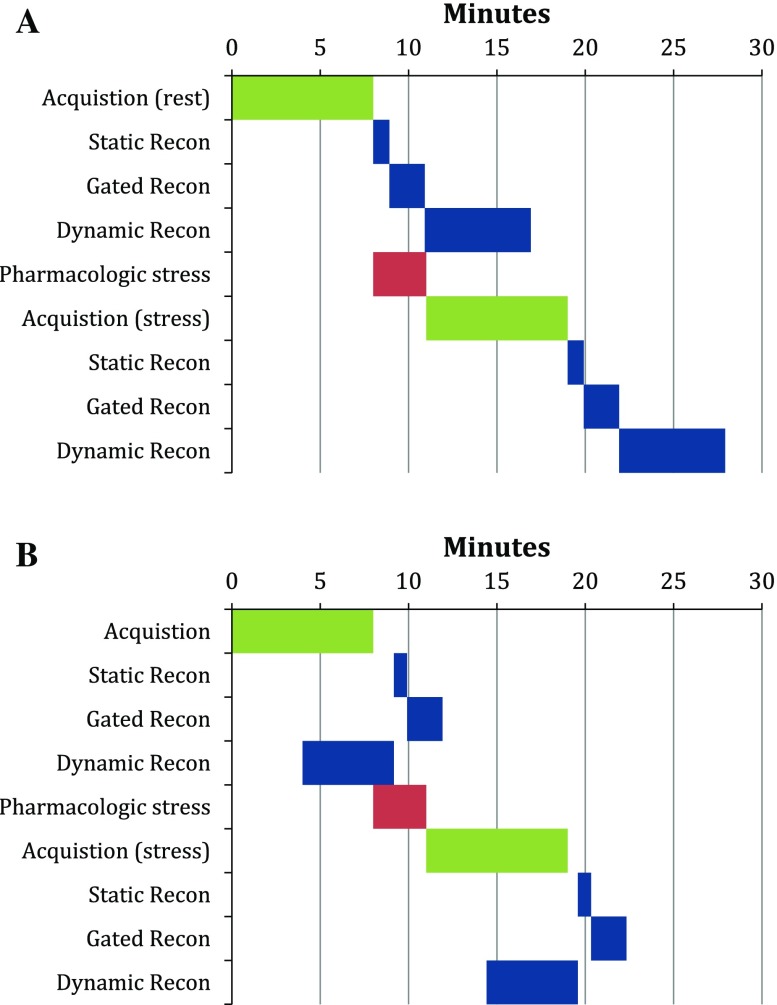
**A** Serial PET reconstruction that starts after the PET acquisition does not complete image reconstruction until 9 minutes post acquisition **B** Parallel PET reconstruction that starts in parallel with the PET acquisition completes the image reconstruction in less than 4 minutes post acquisition

In order to optimize image reconstruction and improve signal-to-noise (SNR) ratio, Biograph mCT incorporates detector point spread function (PSF) and ToF into its standard iterative reconstruction algorithms.^12^,^19^–^21^ The serial reconstruction time for all images (static, gated, dynamic), within one PET acquisition, is approximately 9 minutes post acquisition. In an effort to reduce reconstruction times, Siemens Healthineers introduced a software solution that allows parallel processing of PET data. This innovative approach allows the PET data reconstruction process to begin during an active PET acquisition. PET images reconstruct, and are available for viewing, in less than 4 minutes post acquisition. Figures 4A and B show the gain in processing time compared to serial reconstructions.

## Software: Post-Processing

### *syngo*.via

Since today’s PET/CTs produce more complex data than ever before, managing such a volume of information can present challenges. Designed to address these challenges, *syngo*.via is Siemens Healthineers’ integrated multimodality imaging solution. As an open platform it allows the user to extend and customize the interface for an optimal reading experience in cardiac applications as well as oncological, neurological, and general nuclear medicine applications. Siemens Healthineers offers three well-known cardiac post-processing solutions: Corridor4DM,^22^ Cedars Cardiac Suite,^23^ and *syngo.*PET MBF. All seamlessly integrate into the *syngo*.via platform and allow users to compute semi-quantitative and quantitative metrics for MPI. Users may read all Siemens Healthineers’ cardiac PET/CT data on any other industry-standard cardiac post-processing solution. Furthermore, *syngo*.via supports structured reporting (SR) to facilitate information sharing.

### *syngo*.PET MBF

The current practice of managing patients with CAD observed a recent paradigm shift to include functional diagnostic and therapeutic strategies. Several invasive coronary pressure- and flow-measurement options show value in diagnostic and therapeutic decision making. To avoid the increased risk of adverse events that come with the invasive nature of those procedures, a non-invasive technology that allows a comprehensive, physiological assessment of the coronary status could be of utmost clinical relevance. The option to detect diseases from advanced, flow-limiting epicardial CAD to earlier stages of atherosclerosis or microvascular dysfunction has the potential to optimize patient management and risk stratification.^3^,^4^,^24^

The importance of non-invasive quantification of MBF as a parameter to establish an understanding of the physiology of the coronary arteries was recognized about 30 years ago.^25^ Since then, development in scanner instrumentation and software makes it possible to envision a broad clinical use of MBF and MFR measurements that help guide clinical management and advance research. While an ideal perfusion tracer for MBF quantification would have a 100% extraction rate from blood to tissue and no washout from the myocardial cells, Rubidium-82 chloride and Nitrogen-13 ammonia both have a non-linear net-tracer uptake in the myocardium, especially in the higher coronary flow range.^8^,^26^ This uptake leads to an underestimation of the calculated MBF at high flow levels and therefore requires the application of proper physiological compensation algorithms.^26^,^27^ Software programs for MBF and MFR calculation should take into account the non-linear extraction of the clinically available radiopharmaceuticals. Siemens Healthineers was among the first to provide PET/CT cardiology customers with a regulatory-approved MBF application: *syngo*.PET MBF. The application is based on the work of several leading academic institutions who demonstrate the feasibility of MBF and MFR calculations based on dynamic Nitrogen-13 ammonia and Rubidium-82 chloride PET data.^10^,^28^,^29^ The provided solution seamlessly integrates the compartmental kinetic analysis of dynamic PET data into the clinical workflow.

**Figure 5:**
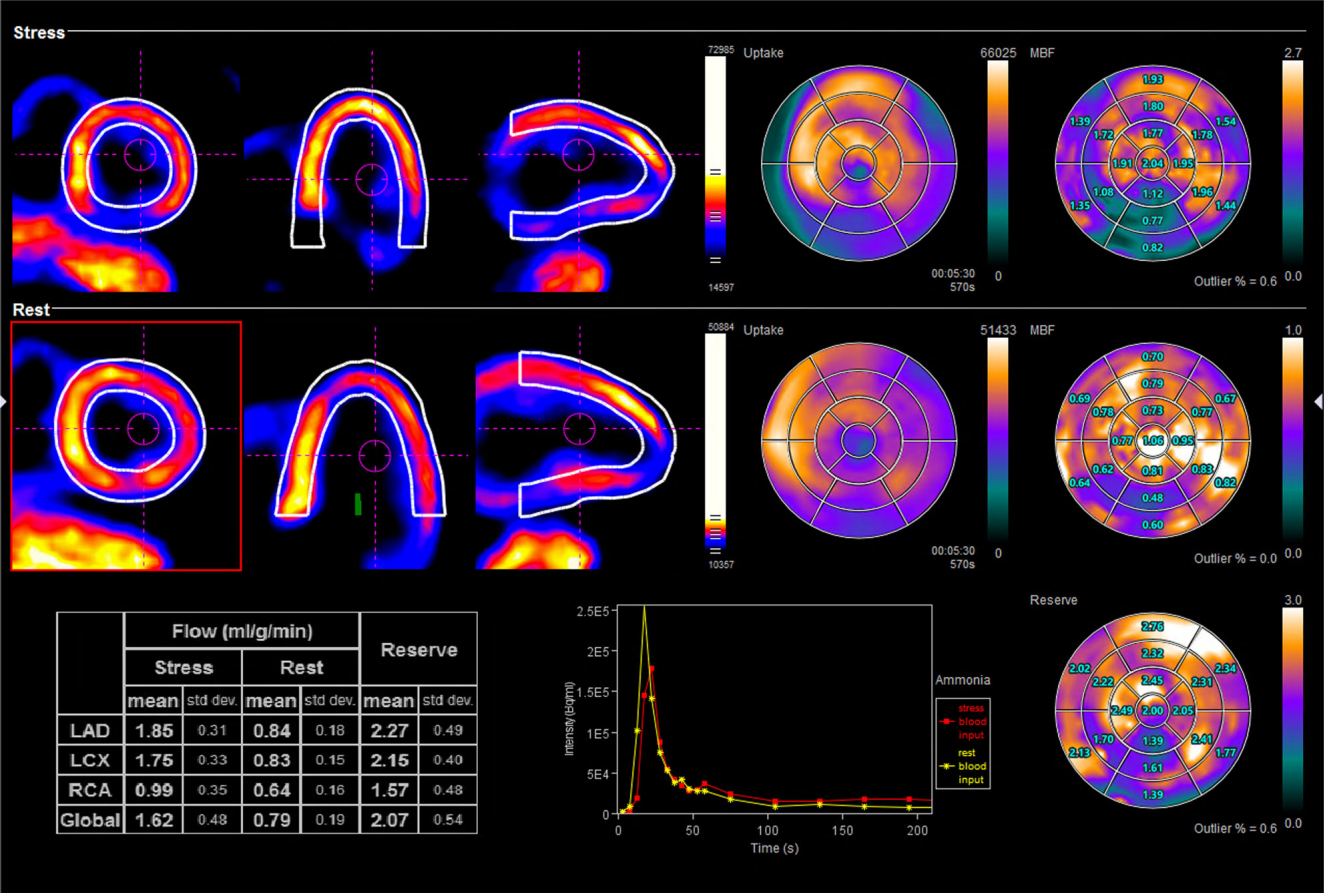
Quantitative assessment of the myocardium using *syngo*.PET MBF. The quantitative MBF values for stress, rest, and MFR display in table form as well as polar plot form with a 17-segment overlay. The blood input function, as well as the stress and rest TAC, display in graphical form

For input, the application uses the reconstructed dynamic stress and rest cardiac Nitrogen-13 ammonia or Rubidium-82 chloride images. The principals of compartmental modeling form the basis of the MBF calculation; *syngo*.PET MBF uses a one-compartment model^30^ for Rubidium-82 chloride and a two-compartment model for Nitrogen-13 ammonia to compensate for the non-linear tracer uptake.^31^ The software automatically applies LV motion correction to the later frames and offers an additional motion correction option in the case of severe motion artifacts. When using Nitrogen-13 ammonia, a first-frame-subtraction method compensates for residual activity from a previous study.^32^ For output, the application provides quantitative and visual information about the perfusion capabilities of the major coronary arteries. The quantitative MBF values for stress, rest, and MFR display in table form; visualization of MBF and MFR displays in polar plot form; and the blood input function, as well as the stress and rest time activity curves (TAC), display in graphical form. The application groups the information by coronary artery territory to aid the physician in the localization of any noted pathology (Figure 5). The software also allows the creation of hospital-specific normal databases.

A multicenter study comparing ten software packages, including *syngo*.PET MBF, concludes that MBF and MFR values computed from Rubidium-82 chloride correlate well when using the one-compartment model.^30^,^33^ A similar study comparing three software tools, including *syngo*.PET MBF, concludes that MBF and MFR values derived from Nitrogen-13 ammonia demonstrate excellent correlation.^34^ Such a conclusion underlines *syngo*.PET MBF’s potential use in both routine clinical settings as well as clinical research projects for Rubidium-82 chloride and Nitrogen-13 ammonia. 33,34

## New Clinical Horizons Using Motion-Free List Mode Data

**Figure 6:**
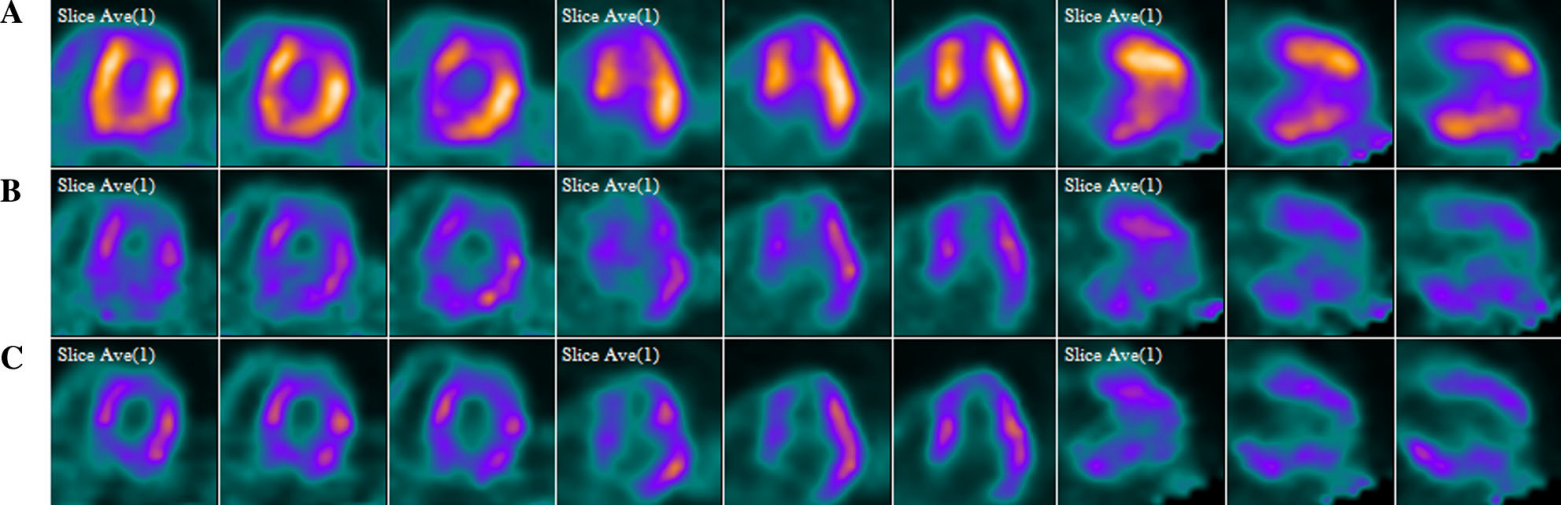
Comparison between **A** static (upper row) **B** cardiac gated (middle row) **C** motion-frozen, gated stress with CardioFreeze (lower row) MPI images. Data courtesy of University of Michigan, Ann Arbor, USA

Given that PET data acquisitions are not instantaneous, PET images often appear blurred as a result of patient motion. Image blurring can impact the visualization of the myocardial tracer distribution or the assessment of wall thickness. Conventional dual gating options use electrocardiogram (ECG) leads and a variety of respiratory gating instrumentation. The latter often requires a time-intensive setup, can cause measurement artifacts, and captures only external body motion as a surrogate for the actual motion of the internal organs. To further expand respiratory and cardiac motion correction, Siemens Healthineers recently introduced their new motion management feature: CardioFreeze. CardioFreeze simultaneously detects and compensates for respiratory motion by analyzing the PET list mode data and applying a deblurring algorithm to gated images based on mass preservation optical flow.^12^,^35^,^36^ Compared to conventional dual gating, CardioFreeze enables each individual image to reconstruct using all counts: up to 24 times the number of counts in each gated image, depending upon the number of gates in the reconstruction (Figure 6). Since the scanner takes 100% of the PET statistics into account there is a possibility of improved image quality and SNR, or a reduction in PET scan time, or injected dose.^37^ Viability assessment, perfusion imaging, and the evaluation of inflammatory processes—such as Sarcoidosis or intravascular infections—are likely to benefit from this feature.

## Conclusion and Outlook

As an innovator in medical imaging, Siemens Healthineers strives to continuously improve existing products and create new solutions that address challenges specific to cardiac imaging. Siemens Healthineers’ Biograph mCT provides innovative, reliable, and easy-to-use capabilities that ensure an optimal quantitative and qualitative PET/CT acquisition and image formation workflow. A seamless integration of post-processing and viewing applications enables the completion of an efficient, high-quality cardiac and PET/CT examination that includes MBF and MFR calculations. Once clinical practices further integrate MPI and MBF assessments into their routine and Fluorine-18 labeled perfusion tracers receive regulatory approval, the resulting physiological information can apply to many additional clinical scenarios. Such scenarios include the management of heart failure, detailed assessment of coronary vasodilator function, and monitoring of coronary endothelial function in response to statin therapy.^3^ As for the future of PET/CT technology, Siemens Healthineers’ innovative Biograph Vision^1^ recently introduced the Optiso Ultra Dynamic Range (UDR) detector. The detector’s unique design enables high spatial resolution, increased sensitivity, and an unmatched temporal resolution.^38^ By complementing the effects of the reduced dead time with high sensitivity and resolution, this new detector intends to capture more image detail in single-injection protocols for MBF and MPI assessment. Siemens Healthineers’ Biograph family, in combination with *syngo.*via, continually evolves to meet emerging clinical needs and offers effective tools to assess a wide spectrum of CAD.

## Electronic supplementary material

Below is the link to the electronic supplementary material.

Supplementary material 1 (PPTX 1968 kb)

## Abbreviations

PET/CT: Positron emission tomography, combined with computed tomography
MBF: Myocardial blood flow
MFR: Myocardial flow reserve
BIF: Blood input function
MPI: Myocardial perfusion imaging
CAD: Coronary artery disease
LSO: Lutetium oxyorthosilicate
AC: Attenuation correction
PGC: Prompt gamma correction
SMART: Siemens molecular & anatomical registration technologies

## Footnotes

1 Biograph Vision is currently under development and is not yet commercially available in the European Union, the U.S. or any other country.
