# Supplementary material for: The continual innovation of commercial PET/CT solutions in nuclear cardiology: Siemens Healthineers
Source: J Nucl Cardiol. 2018 Apr 10;25(4):1400–11. doi: 10.1007/s12350-018-1262-3 (PMC6133132; doi:10.1007/s12350-018-1262-3)
Supplement: Supplementary file 1 — Supplementary material 1 (PPTX 1968 kb) [file 12350_2018_1262_MOESM1_ESM.pptx]

## Slide 1
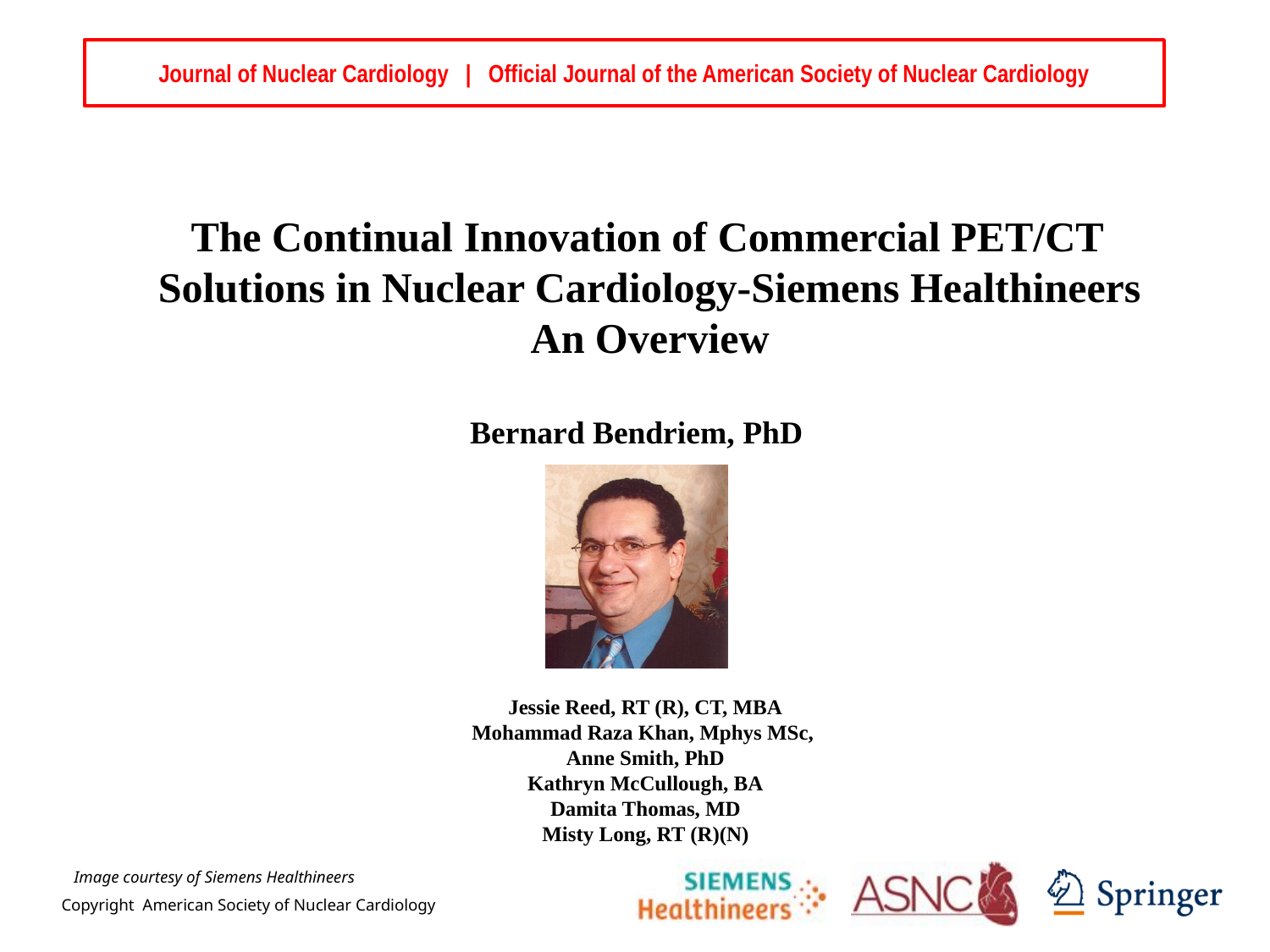

Journal of Nuclear Cardiology | Official Journal of the American Society of Nuclear Cardiology
# The Continual Innovation of Commercial PET/CT Solutions in Nuclear Cardiology-Siemens Healthineers An Overview
Bernard Bendriem, PhD
Jessie Reed, RT (R), CT, MBA
Mohammad Raza Khan, Mphys MSc,
Anne Smith, PhD
Kathryn McCullough, BA
Damita Thomas, MD
Misty Long, RT (R)(N)
 Image courtesy of Siemens Healthineers
Copyright American Society of Nuclear Cardiology

## Slide 2
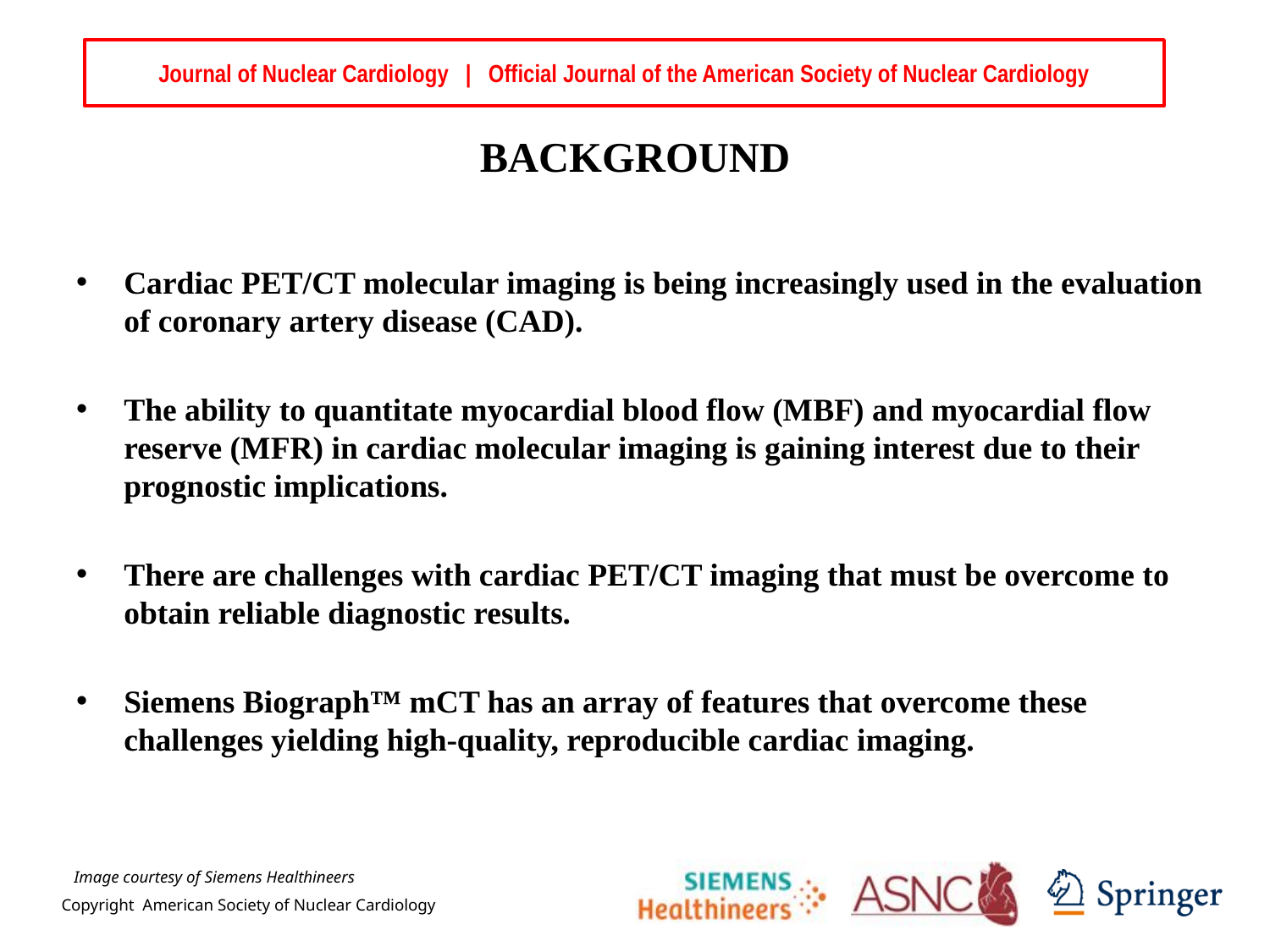

Journal of Nuclear Cardiology | Official Journal of the American Society of Nuclear Cardiology
# BACKGROUND
Cardiac PET/CT molecular imaging is being increasingly used in the evaluation of coronary artery disease (CAD).
The ability to quantitate myocardial blood flow (MBF) and myocardial flow reserve (MFR) in cardiac molecular imaging is gaining interest due to their prognostic implications.
There are challenges with cardiac PET/CT imaging that must be overcome to obtain reliable diagnostic results.
Siemens Biograph™ mCT has an array of features that overcome these challenges yielding high-quality, reproducible cardiac imaging.
 Image courtesy of Siemens Healthineers
Copyright American Society of Nuclear Cardiology

## Slide 3
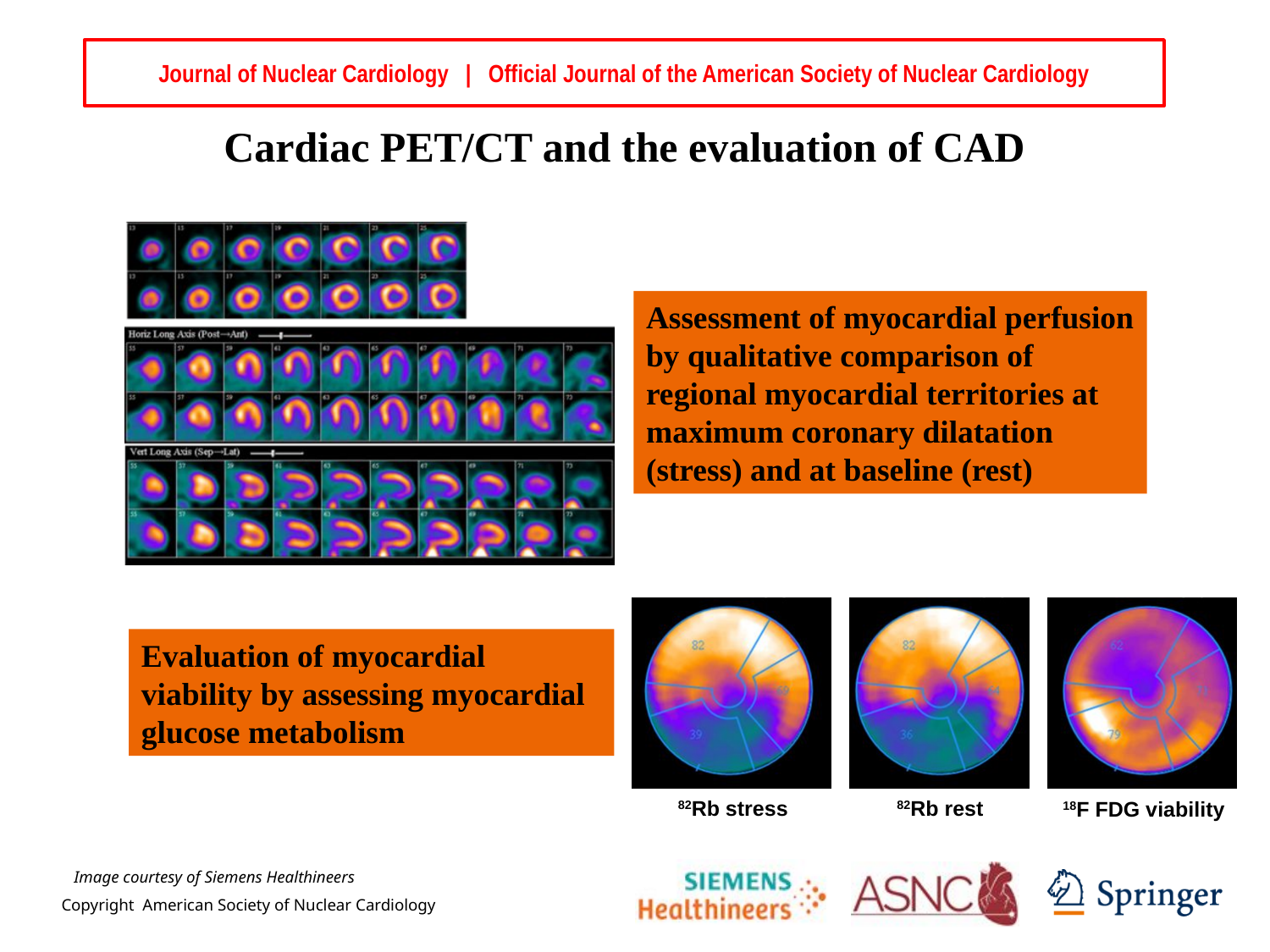

Journal of Nuclear Cardiology | Official Journal of the American Society of Nuclear Cardiology
# Cardiac PET/CT and the evaluation of CAD
Assessment of myocardial perfusion by qualitative comparison of regional myocardial territories at maximum coronary dilatation (stress) and at baseline (rest)
Evaluation of myocardial
viability by assessing myocardial glucose metabolism
82Rb rest
82Rb stress
18F FDG viability
 Image courtesy of Siemens Healthineers
Copyright American Society of Nuclear Cardiology

## Slide 4
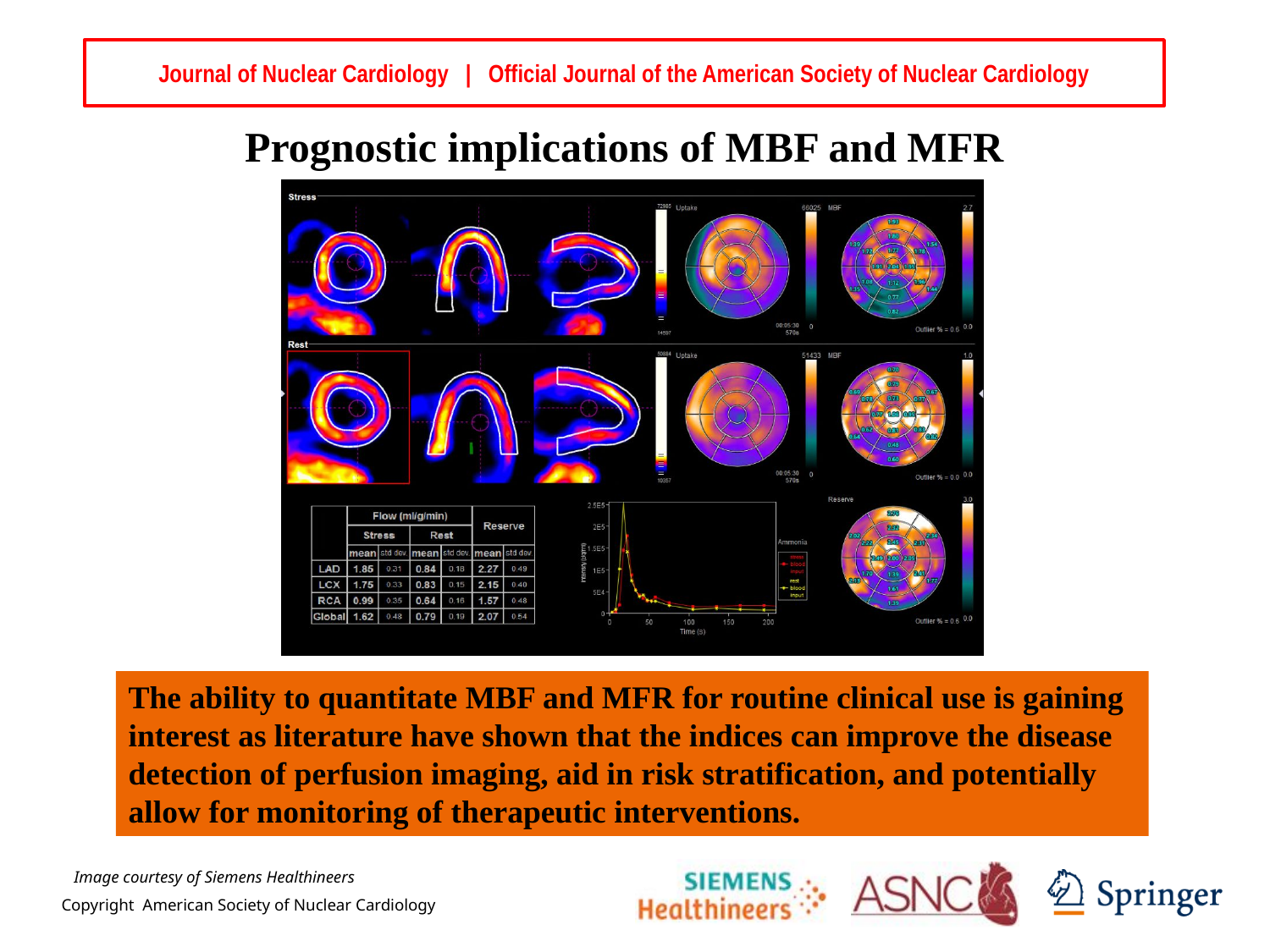

Journal of Nuclear Cardiology | Official Journal of the American Society of Nuclear Cardiology
# Prognostic implications of MBF and MFR
The ability to quantitate MBF and MFR for routine clinical use is gaining interest as literature have shown that the indices can improve the disease detection of perfusion imaging, aid in risk stratification, and potentially allow for monitoring of therapeutic interventions.
 Image courtesy of Siemens Healthineers
Copyright American Society of Nuclear Cardiology

## Slide 5
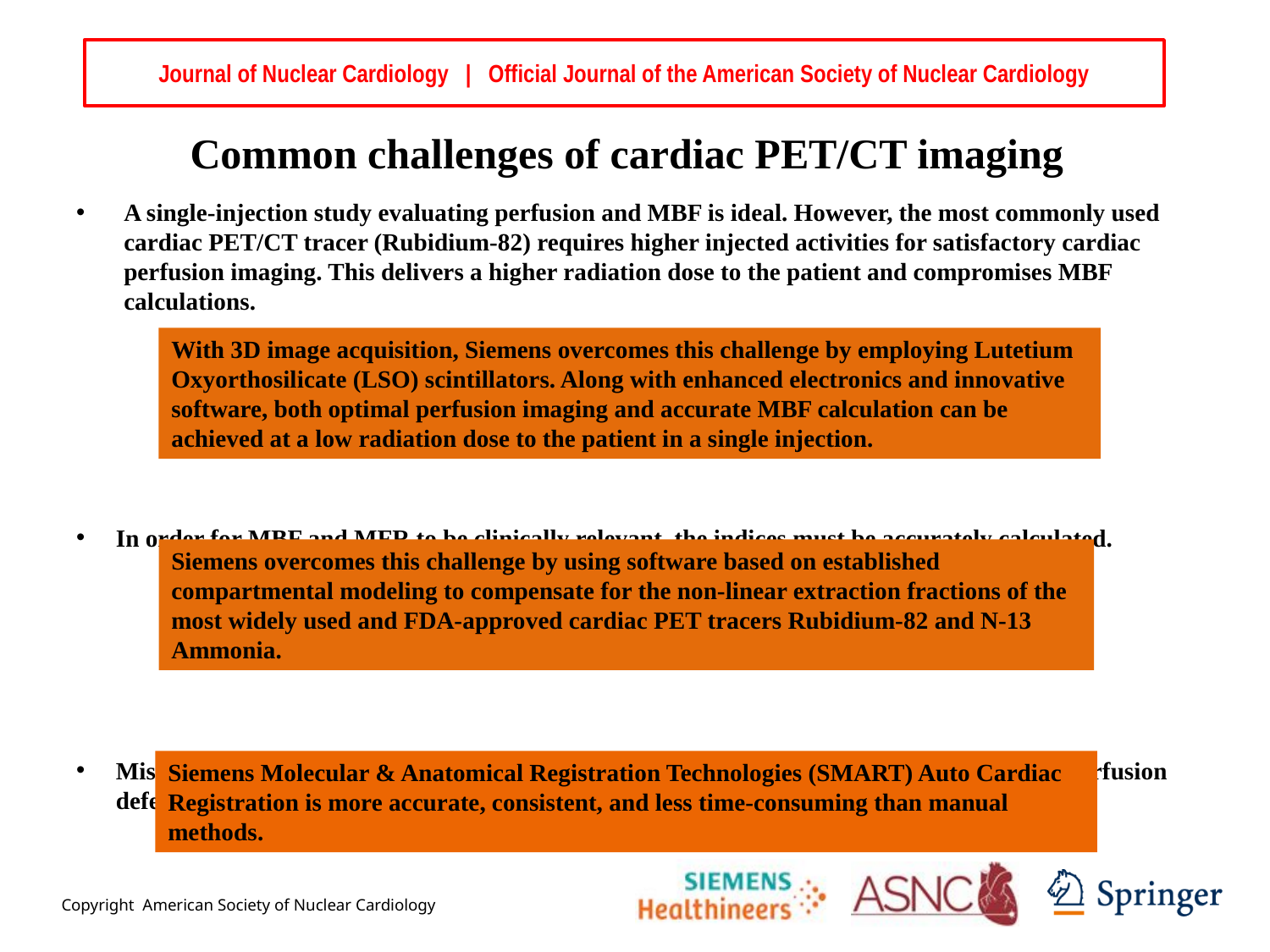

Journal of Nuclear Cardiology | Official Journal of the American Society of Nuclear Cardiology
# Common challenges of cardiac PET/CT imaging
A single-injection study evaluating perfusion and MBF is ideal. However, the most commonly used cardiac PET/CT tracer (Rubidium-82) requires higher injected activities for satisfactory cardiac perfusion imaging. This delivers a higher radiation dose to the patient and compromises MBF calculations.
In order for MBF and MFR to be clinically relevant, the indices must be accurately calculated.
Misregistration between functional PET and anatomical CT images can create artifactual perfusion defects.
With 3D image acquisition, Siemens overcomes this challenge by employing Lutetium Oxyorthosilicate (LSO) scintillators. Along with enhanced electronics and innovative software, both optimal perfusion imaging and accurate MBF calculation can be achieved at a low radiation dose to the patient in a single injection.
Siemens overcomes this challenge by using software based on established compartmental modeling to compensate for the non-linear extraction fractions of the most widely used and FDA-approved cardiac PET tracers Rubidium-82 and N-13 Ammonia.
Siemens Molecular & Anatomical Registration Technologies (SMART) Auto Cardiac Registration is more accurate, consistent, and less time-consuming than manual methods.
Copyright American Society of Nuclear Cardiology

## Slide 6
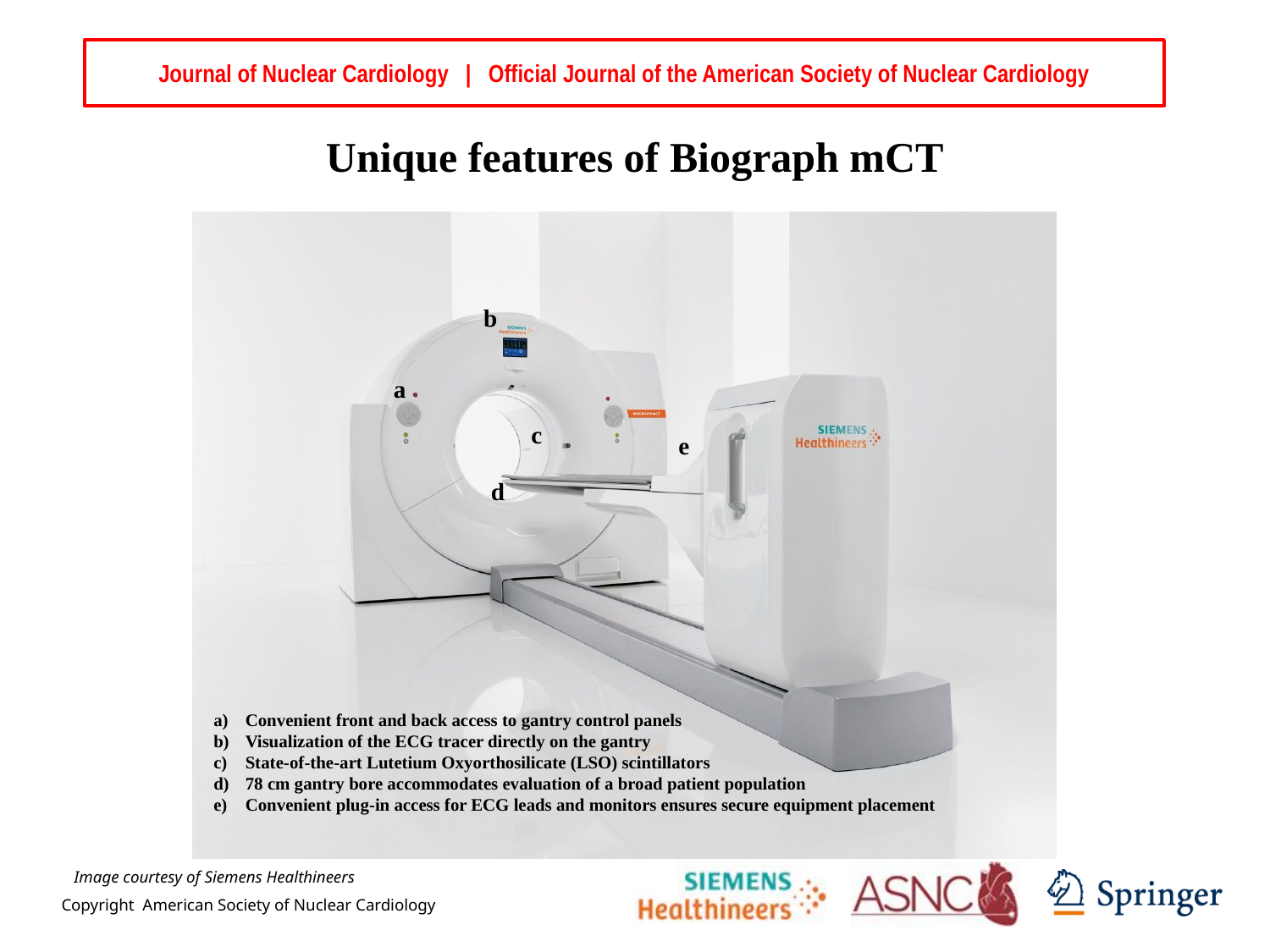

Journal of Nuclear Cardiology | Official Journal of the American Society of Nuclear Cardiology
# Unique features of Biograph mCT
b
a
c
e
d
Convenient front and back access to gantry control panels
Visualization of the ECG tracer directly on the gantry
State-of-the-art Lutetium Oxyorthosilicate (LSO) scintillators
78 cm gantry bore accommodates evaluation of a broad patient population
Convenient plug-in access for ECG leads and monitors ensures secure equipment placement
 Image courtesy of Siemens Healthineers
Copyright American Society of Nuclear Cardiology

## Slide 7
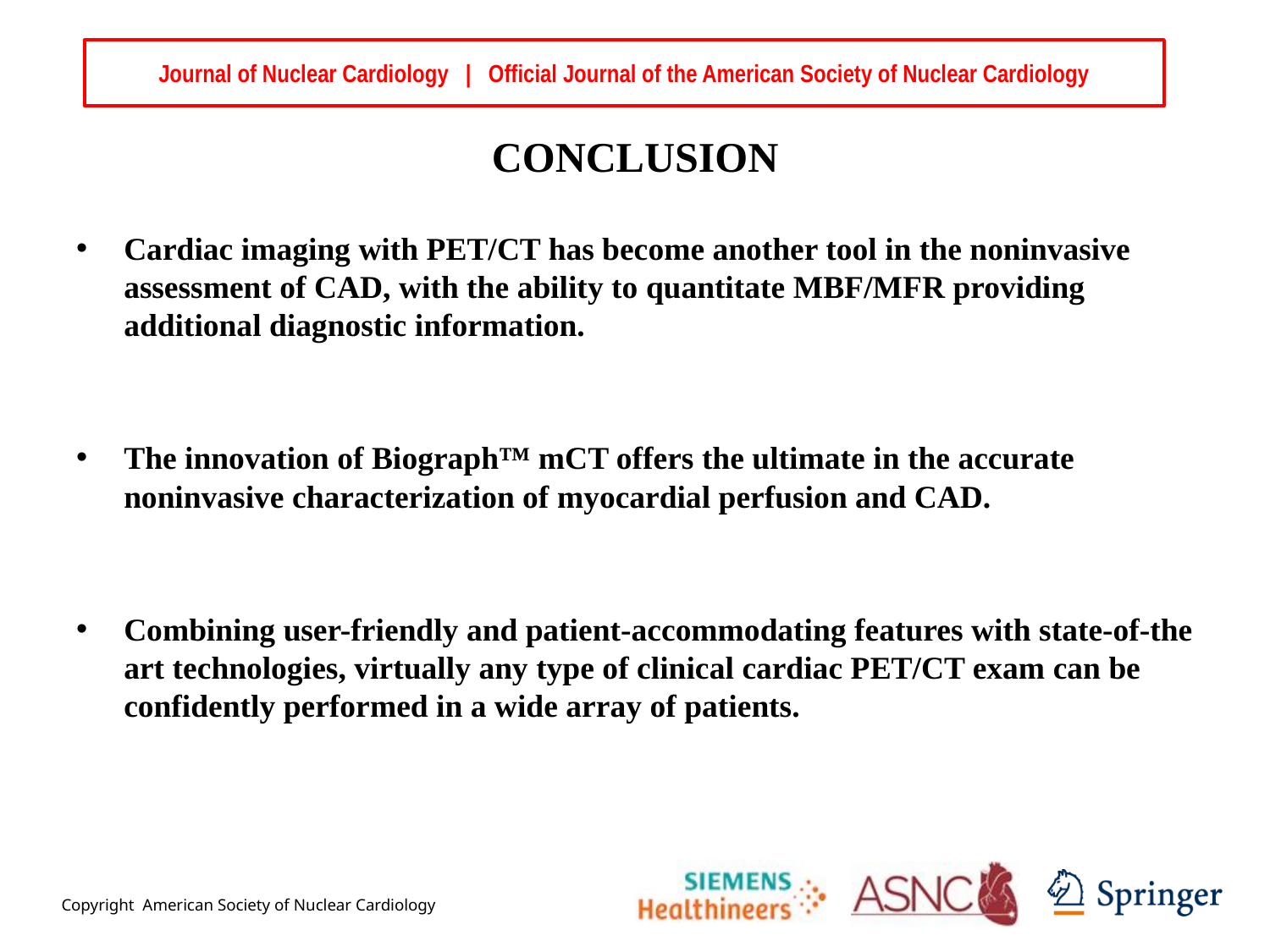

Journal of Nuclear Cardiology | Official Journal of the American Society of Nuclear Cardiology
# CONCLUSION
Cardiac imaging with PET/CT has become another tool in the noninvasive assessment of CAD, with the ability to quantitate MBF/MFR providing additional diagnostic information.
The innovation of Biograph™ mCT offers the ultimate in the accurate noninvasive characterization of myocardial perfusion and CAD.
Combining user-friendly and patient-accommodating features with state-of-the art technologies, virtually any type of clinical cardiac PET/CT exam can be confidently performed in a wide array of patients.
Copyright American Society of Nuclear Cardiology
